# Emotional Modulation of Attention: Fear Increases but Disgust Reduces the Attentional Blink

**DOI:** 10.1371/journal.pone.0007924

**Published:** 2009-11-19

**Authors:** Nicolas Vermeulen, Jimmy Godefroid, Martial Mermillod

**Affiliations:** 1 Psychology Department, Université catholique de Louvain (UCL), Louvain-la-Neuve, Belgium; 2 Laboratoire de Psychologie Sociale et Cognitive (LAPSCO), Université Blaise Pascal, Clermont-Ferrand, France; Victoria University of Wellington, New Zealand

## Abstract

**Background:**

It is well known that facial expressions represent important social cues. In humans expressing facial emotion, fear may be configured to maximize sensory exposure (e.g., increases visual input) whereas disgust can reduce sensory exposure (e.g., decreases visual input). To investigate whether such effects also extend to the attentional system, we used the “attentional blink” (AB) paradigm. Many studies have documented that the second target (T2) of a pair is typically missed when presented within a time window of about 200–500 ms from the first to-be-detected target (T1; i.e., the AB effect). It has recently been proposed that the AB effect depends on the efficiency of a gating system which facilitates the entrance of relevant input into working memory, while inhibiting irrelevant input. Following the inhibitory response on post T1 distractors, prolonged inhibition of the subsequent T2 is observed. In the present study, we hypothesized that processing facial expressions of emotion would influence this attentional gating. Fearful faces would increase but disgust faces would decrease inhibition of the second target.

**Methodology/Principal Findings:**

We showed that processing fearful versus disgust faces has different effects on these attentional processes. We found that processing fear faces impaired the detection of T2 to a greater extent than did the processing disgust faces. This finding implies emotion-specific modulation of attention.

**Conclusions/Significance:**

Based on the recent literature on attention, our finding suggests that processing fear-related stimuli exerts greater inhibitory responses on distractors relative to processing disgust-related stimuli. This finding is of particular interest for researchers examining the influence of emotional processing on attention and memory in both clinical and normal populations. For example, future research could extend upon the current study to examine whether inhibitory processes invoked by fear-related stimuli may be the mechanism underlying the enhanced learning of fear-related stimuli.

## Introduction

Facial emotions are known to be central for social communication[1]. Research shows that the ability to identify expressive emotional actions can affect the social interaction of normal populations[2] and those with psychopathology[3]–[5]. This is because facial expressions communicate the intentions, needs and emotional states of the senders (expressers), and, as such, receivers (observers) are required to quickly and efficiently detect these expressions so that they may adjust their behaviors accordingly. The social importance of facial expressions may explain why humans preferentially detect emotional faces based on visual saliency of specific features[6], [7].

Recent neuroimaging and neurophysiological studies have proposed that processing fear expressions is related to increased neural activity in areas involved in attentional networks[8], such that fearful faces enhance visual responses in the extrastriate occipital cortex[9]. Moreover, it has recently been shown that fearful and disgust expressions are not merely arbitrary social signals, but gestures that have physiological consequences[10]. Whereas fearful expressions are associated with a larger visual field, faster eye movements and increased nasal volume and air velocity, the opposite pattern of physiological properties has been found for disgust faces in expressers. In other words, fear and disgust serve to respectively increase versus diminish sensory interactions with the environment.

One important issue relates to the way observers process, understand and utilize facial cues. It has been suggested that the sensory benefit of expressed emotions in the senders is transmitted to receivers under the form of preparatory action tendencies[10]. This explains why observers have facial reactions to facial expressions[11]. The way observers understand expressers partly depends on their ability to mirror the emotional states of others in themselves through a simulation (i.e., mirroring) process which is thought to represent the (neural) basis of social sharing of emotional states[12]–[14]. Emotional representation depends on the ability to simulate emotions in oneself[15]–[18]. This theory is used to explain neuroimaging results which showed that the same areas of the anterior insula (known as a gustatory cortex) and ACC constitute a shared neural basis of seeing and feeling disgust[14]. Based on this hypothesis, in the present study, we assumed that these configured properties of fearful and disgust expressions might have a similar influence on the attentional capacities of observers. Therefore, based on both this simulation theory[15]–[18] and on the differential role of fear and disgust on physiological properties[10], we hypothesized that fear would boost attentional resources, whereas disgust would reduce the availability of attentional resources in observers.

In order to test whether fear and disgust facial expressions are configured to enhance versus diminish the attention of observers, we used the AB paradigm. In rapid serial visual presentation (RSVP, with up to 19 items per second), AB refers to the negative effect of the first target (T1) on the second target (T2) identification within a period of 200–500 ms following T1[19]–[21]. The AB paradigm is one of the most widely used paradigms to study the time course of visual attention.

Different theoretical frameworks have been proposed to account for the AB. On the one hand, a theory called “two-stage competition model of attention[20]” proposes that AB is caused by capacity limitation. This model proposes that in Stage 1, T1 is detected on the basis of some relevant features (e.g., being a word) and grabs attentional resources used to complete its full (lexical) identification. Since it takes 50–100 ms for T1 to be identified, if T2 appears during this stage, it will compete for resources while the two targets are in stage 1 (i.e., typical AB effect). At very short SOAs (13 to 53 ms), T2 benefits from the prior capture of attention by T1 and will be likely identified first (an effect called ‘lag-1 sparing’). As a result, the target that is first identified enters stage 2, where it will be consolidated into short-term memory (STM), during the first 200–500 ms. On the other hand, it was recently proposed that the AB reflects rather a selection deficit that is not caused by capacity limitation[19]. This recent “boost and bounce” (B&B) theory of temporal attention proposes that AB and lag-1 sparing effects are related to the presence of a gating system that promotes the entrance of relevant information (target-like features) or prevents the entrance of irrelevant information (distractor-like features) into working memory[19]. During the sensory processing stage, representations of perceptual features or semantic information are activated. The second stage relates to working memory, which serves to monitor, maintain and report information. Between these two stages, gate neurons provide excitatory or inhibitory feedback responses. Such feedback responses could depend on the norepinephrine release under the control of the locus coeruleus[22]. When a stimulus matches the target-like description, an excitatory feedback activity is triggered (a “boost”) favoring the access to working memory. Following distractors, a strong inhibitory feedback response (a “bounce”) closes the gate to working memory, resulting in a subsequent AB in which T2 suffers from the inhibition of distractors. The lag-1 sparing effect is also explained by this model because T2 arrives in the peak of the attentional boost that follows T1 processing. As a result, T2 is easily processed and often masks T1.

Based on this theory, we hypothesized that emotion might interfere with the AB: fear should maximize these boost and bounce effects whereas disgust should minimize them. During the typical AB, T2 should be less blinked (i.e., better reported) following the processing of a disgusted face (which diminishes allocation of attentional resources) than following a fearful face (which enhances allocation of attentional resources). Such results would provide strong evidence that fear and disgust might distinctively boost or bounce attentional gating to working memory in observers. In the present study, each RSVP trial involving two target words (T1/T2) was preceded by the processing of either a fear or a disgust face.

## Methods

The participants were 18 French speakers (9 females; Age: *M = *19.56; *SD = *2.6) who were tested individually and paid 5€. The ethical committee of the University of Louvain approved the protocol and informed written consent was obtained from the subjects after the nature and possible consequences of the study were explained to them. All had normal or corrected-to-normal vision. Forty pairs of targets (T1-T2) were created using 4 to 7 letters words. The two targets, matched for length, always appeared in a same stream of stimuli (one stimulus replacing another). The distractors were comprised of random strings of symbols and digits of the same length as the words on each trial. Thirty-two faces of eight actors (4 females), each portraying Fear, Disgust, Sadness and Joy were selected from the Montreal Set of Facial Displays of Emotion (MSFDE)[23]. Target words appeared in uppercase letters. All stimuli (distractors and targets) were black, presented on a white background, and all words were presented in Courier New 18-point bold font. The three independent variables were prime face type (Fear or Disgust), SOA (53 or 213), and target type (T1 or T2). A within-subject design was adopted.

Stimuli were presented using E-Prime 1.1.4.1 on Dell PC with Processor Intel-Pentium IV 2.3 GHz/256Mb SDRAM computer with a 17-in. monitor with a refresh rate of 75 Hz. The participants read computer-presented instructions. The task was to detect and report (using the keyboard) the two words that appeared among the distractors. There were a total of 40 trials. The presentation duration was set to 53 ms/item.

**Figure 1:**
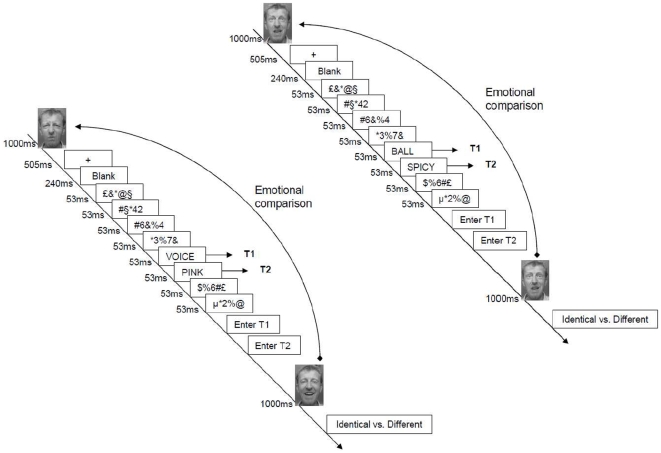
Schematic overview of typical trials with a stimulus onset asynchrony (SOA) of 53 milliseconds. Each trial started with the presentation of a facial expression directly followed by the rapid serial visual presentation (RSVP). Each stimulus was presented one at a time in the center of the screen for 53 ms. After the participants entered the target words (T1 and T2) they saw, they made a emotional comparison judgement (identical vs different) of the second face (different on the left sequence and identical on the right sequence). For the need of this figure, the pictures were not taken from the MSFDE as in the experiment but represent the first author of the paper (N.V.).

As shown in Fig. 1, each trial started with the presentation of a to-be assessed facial expression (Fear or Disgust). We will refer to this facial expression as the “prime”. Then followed the RSVP of distractors and targets. The stimulus onset asynchrony (SOA) between the two target words was set to 53 ms (no distractor) or 213 ms (three distractors). After each RSVP, participants were instructed to type the first word they saw in the RSVP trial (T1), followed by the second word they saw (T2). Then, they saw a second facial expression they had to evaluate as expressing an identical or a different emotional expression compared to the first face they saw. The 16 Fear and Disgust faces that served as prime faces were also presented during the identical vs. different decisions stage, whereas the 16 Sad and Joy faces served only as different facial expression stimuli during the identical vs. different decision stage. All the faces were randomly assigned and repeated depending on their condition.

There were three to five distrators before the presentation of T1, and T2 was always followed by two distractors. Within a trial, the same distractor was never repeated. The next trial started 1000 ms after the decision on the second face (Identical versus Different Emotion) was made.

An analysis of variance (ANOVA) was used to analyse the accuracy of identifying the targets T1 and T2. Misspelled words and blanks were counted as errors[24]. Mean percentages of correct report for T1 and T2 (contingent upon T1 correct) were calculated, with order reversals counted as correct[19].

## Results and Discussion

**Figure 2:**
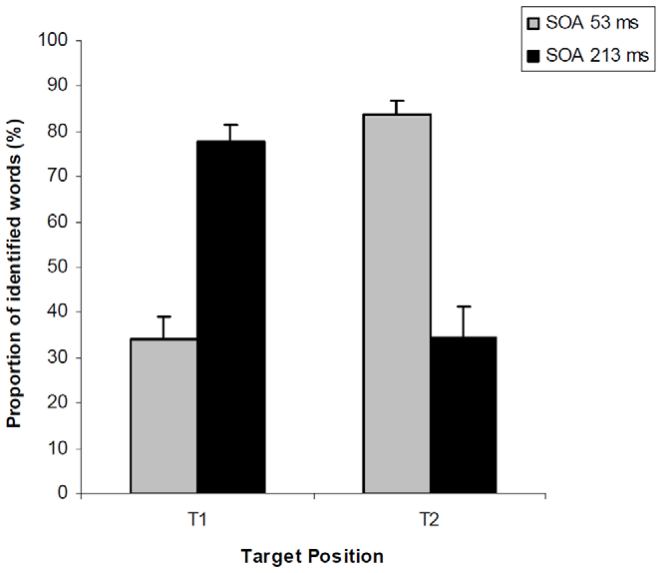
The ability to detect a target word depends on the target position (T1 and T2) and the time separating the onset of the two targets (SOA). Paired *t*-tests comparisons showed that when the stimulus onset asynchrony (SOA) is short (53 ms) T2 is better reported than T1 (*t*_17_ *= *12.56, *P*<.001). On the reverse, when the SOA is longer (213 ms, 3 distractors between T1 and T2), T1 is better reported than T2 (*t*_17_ *= *7.47, *P*<.001). Error bars indicate s.e.m.

Overall, 85.3% of the facial expressions were correctly identified and 57.4% of the words were reported accurately, which corresponds to slightly more than one word per trial (on two possible words). As shown in Fig. 2 and consistent with previous studies[20], [24], [25], there was a significant crossover interaction between SOA and Target position (*F*_1,17_ *= *140.96, *P*<0.001). For the long SOA, there was a large AB effect, with T1 reported more accurately than T2. However, for the short SOA, there was a lag-1 sparing effect, with T2 better reported than T1.

Our main question was whether the AB would be moderated by the prior evaluation of a fear or a disgust prime expression. Even if there was no main effect of emotion prime (*F*_1,17_ *= *1.89, *P = *.19), depending on the SOA, fear and disgust primes distinctively interfered with the identification of words. There was an interaction between Emotion prime and SOA (*F*_1,17_ *= *14.58, *P = *.001) which was mainly driven by the influence of the prime for the longer SOA.

**Figure 3:**
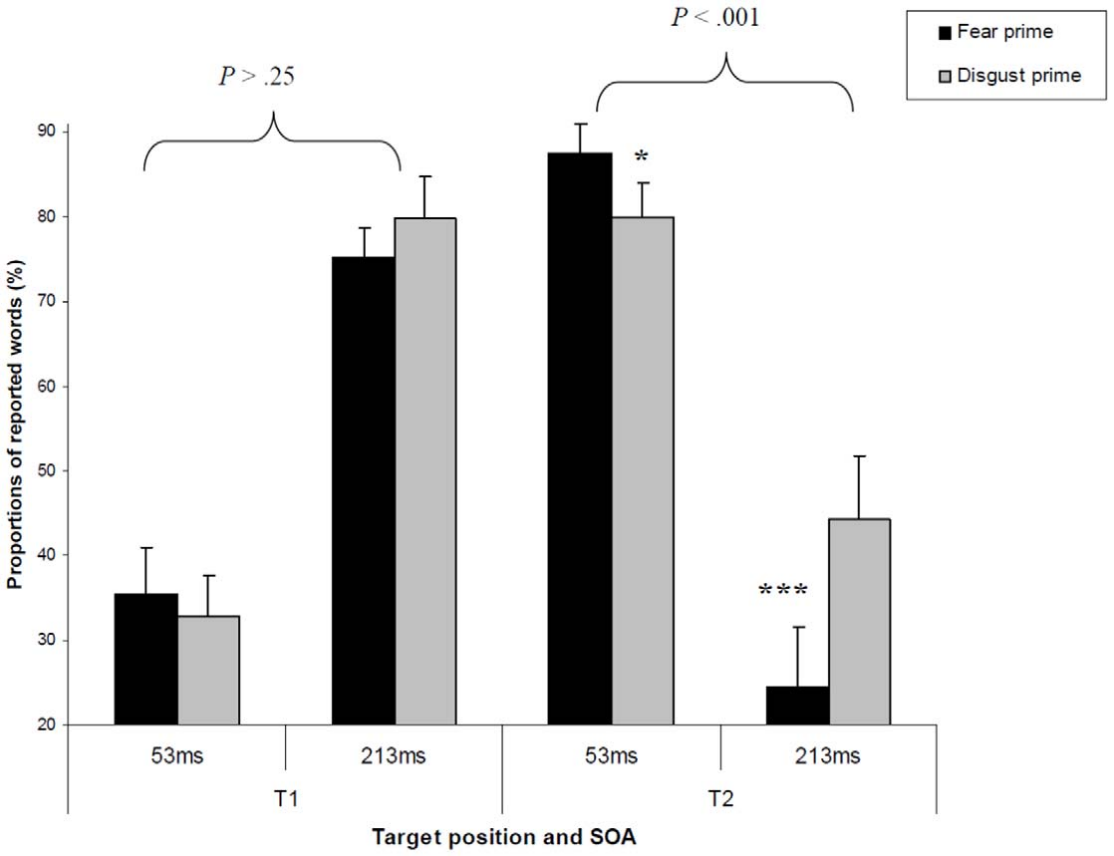
The influence of the emotion prime on target detection depends on the stimulus onset asynchrony (SOA), and specifically for T2 report. The report of T2 targets (not for T1) is better if preceded by a disgust face than by a fear face. Specific comparisons (paired *t*-tests) showed that this effect on T2 identification was mainly driven by the influence of the prime at the longer SOA (213 ms). A significant (*t*_17_ *= *−4.50, *P*<.001) decrease (−19.9%) in correct identification of targets was observed following the processing of fear primes as compared to disgust primes. Whereas at shorter SOA (53 ms), the facilitatory influence (+7.5%) of fear primes on identification was only marginally significant (*t*_17_ *= *1.81, *P*<.09). Error bars indicate s.e.m. Note. * *p*<.10; *** *p*<.001

Of particular interest, the three-way interaction involving Emotion prime, SOA and Target was significant (*F*_1,17_ *= *10.73, *P = *.004). As shown in Fig. 3, the interaction between Emotion prime and SOA was significant for T2 identification only (*F*_1,17_ *= *33.51, *P*<.001) but not for T1 identification (*F*_1,17_ *= *1.40, *P*>.25). In other words, for the longer SOA (i.e., blink) the participants were less able to accurately report the second target word when this second target followed the emotional evaluation of a fear expression compared to a disgust expression. Because SOA was found to influence the target report as a function of the target position, we further decomposed this interaction by analysing separately the Emotion prime by Target in the two SOA conditions. The results showed that in the short SOA condition (i.e., 53 ms), the interaction between Emotion prime and Target was not significant (*F*_1,17_<1, ns). Importantly, in the long SOA condition (i.e., 213 ms), the interaction between Emotion prime and Target was significant (*F*_1,17_ *= *12.95, *P = *.002). This interaction shows that Emotion prime significantly influences T2 report but not T1 report at an SOA of 213 ms (see Fig. 3).

These results provide important information on how attention might be distinctly influenced by those perceived emotions depending on the stimulus onset asynchrony (time-locking). Our findings have implications beyond the simple question of the physiological influence of fear and disgust on sensory exposure in expressers[10]. We showed that, in the perceivers, the central nervous system (generating attentional processes) is also subject to similar influences of emotion processing. Whereas previous behavioural and physiological studies found an effect of perceived fear on brain attentional networks[8] or on sensory exposure[26] (compared to neutral expressions), we found that this effect was reversed for disgust faces. We found that processing fear faces impaired the detection of T2 to a greater extent than did the processing disgust faces. Second, the interaction between emotion prime, target position and SOA support the recent B&B model of attention[19]. Contrary to previous models, B&B suggests that the AB is caused by a too strong attentional response on distractors, and that it is time-locked on the appearance of the first non target-like stimuli (i.e., a distractor that induces a marked inhibitory response). In other words, it is because distractors receive a strong inhibition (i.e., a bounce) that T2 becomes blinked. It is therefore interesting to observe that our results mainly appear on typically missed targets (T2 for the long SOA). Our data suggest that fear causes a stronger attentional modulation and produces a bigger bounce than disgust. A bigger bounce following the processing of fearful expression is consistent with findings showing that threatening information processing is associated with a narrowing of attention to targets and greater inhibition of distractors[27]. Our findings are also consistent with data showing that participants' positive mood influences cognition by decreasing the attentional blink[28]. It might therefore be interesting to examine whether the mere processing of happy faces would also influence RSVP search by decreasing the blink.

Importantly, some limitations and alternative explanations of our results can be entertained. For instance, it could be argued that our findings might also be accounted for by limited capacity models of attention. From that theoretical standpoint, it could still be suggested that fear increases attentional investment on T1, which in turn may cause better sparing, followed by a deeper blink. However, it can be stressed that since there was no sign of T1 detection improvement during our experiment, the B&B model of attention better accounts for our results. Moreover, since we used only two different SOAs further studies may examine whether this effect depends on the time separating the appearance of the targets (i.e., SOA) by using a larger range of lags. This should be interesting since the exact time course of the attentional blink remains unclear. This influence of the SOA may also explain our findings at the short SOA where T2 potentially masked T1. This is indeed likely because both targets were presented in the same visual stream which is known to generate more masking of T1 by T2 at a short SOA than by a distractor at a longer SOA[19]. Future studies could be designed to avoid this problem by using two different streams of stimuli (i.e., one above the other) as was done recently[24]. Another limitation of the present study is related to the absence of a baseline face condition (i.e., neutral). As a result, it is impossible to know if the effects reflect an increased blink following the processing of the fearful face, or to a reduced blink related to the processing of the disgust face. Based on the physiological findings of Susskind and colleagues[10], another possibility is that our findings are related to either an increased blink due to perceived fear or to a decreased blink due to perceived disgust. This possibility seems likely since in a similar design without facial expressions judgment (i.e., two target words in an AB paradigm using the same SOAs), we found that our participants reported T2 in the same long SOA (213 ms) with an accuracy rate (i.e., 33%) that fell just in the middle of the rates we found in the present study following the processing of a fear face (i.e., 24.5%) and the processing of a disgust face (i.e., 44.3%)[25].
